# RhoG’s Role in T Cell Activation and Function

**DOI:** 10.3389/fimmu.2022.845064

**Published:** 2022-02-25

**Authors:** Ana Masara Ahmad Mokhtar, Nor Hawani Salikin, Aminah Suhaila Haron, Syafinaz Amin-Nordin, Ilie Fadzilah Hashim, Muaz Mohd Zaini Makhtar, Siti Balqis Zulfigar, Nurul Izza Ismail

**Affiliations:** ^1^Bioprocess Technology Division, School of Industrial Technology, Universiti Sains Malaysia, Gelugor, Malaysia; ^2^Faculty of Dentistry, AIMST University, Bedong, Malaysia; ^3^Department of Medical Microbiology, Faculty of Medicine and Health Sciences, Universiti Putra Malaysia, Serdang, Malaysia; ^4^Department of Clinical Medicine, Advanced Medical and Dental Institute, Universiti Sains Malaysia, Kepala Batas, Malaysia; ^5^Fellow of Center for Global Sustainability Studies, Universiti Sains Malaysia, Gelugor, Malaysia; ^6^School of Biological Sciences, Universiti Sains Malaysia, Gelugor, Malaysia

**Keywords:** Small Rho GTPase, RhoG, T cell, cancer, hemophagocytic lymphohistiocytosis

## Abstract

The role of RhoG in T cell development is redundant with other Racs subfamily members, and this redundancy may be attributed to redundant signal transduction pathways. However, the absence of RhoG increases TCR signalling and proliferation, implying that RhoG activity is critical during late T cell activation following antigen–receptor interaction. Moreover, RhoG is required to halt signal transduction and prevent hyper-activated T cells. Despite increase in TCR signalling, cell proliferation is inhibited, implying that RhoG induces T cell anergy by promoting the activities of transcription factors, including nuclear factor of activated T cell (NFAT)/AP-1. The role of NFAT plays in T cell anergy is inducing the transcription of anergy-associated genes, such as IL-2, IL-5, and IFN-γ. Although information about RhoG in T cell-related diseases is limited, mutant forms of RhoG, Ala151Ser and Glu171Lys have been observed in thymoma and hemophagocytic lymphohistiocytosis (HLH), respectively. Current information only focuses on these two diseases, and thus the role of RhoG in normal and pathological circumstances should be further investigated. This approach is necessary because RhoG and its associated proteins represent prospective targets for attack particularly in the therapy of cancer and immune-mediated illnesses.

## 1 Introduction

RhoG belongs to the Rho family of small GTPases, specifically the Rac subfamily. The Rho family is involved in actin–cytoskeletal rearrangements, intracellular membrane trafficking, cell cycle progression, and transcriptional activation (1, 2). According to their sequence similarity and biological roles, the Rho family can be divided into the Rho-, Rac-, Cdc42-, RhoU/RhoV-, Rnd-, RhoD/RhoF-, RhoBTB-, and RhoH subfamilies (3). Classical or typical small Rho GTPases are from the Rho-, Rac-, and Cdc42 subfamilies. The other small Rho GTPases are called non-classical or atypical small Rho GTPases because they cannot hydrolyze GTP in contrast to typical small Rho GTPases (4, 5).

Three Rac1, Rac2, Rac3 share 89% sequence similarity, except the C-terminal region (3, 6), whereas RhoG shares only 70%–72% sequence similarity with the other three Racs and may thus act differently within the subfamily. Typically, Rac-subfamily proteins stimulate the formation of membrane ruffles and lamellipodia by interacting with a panel of effector proteins, such as Wiskott–Aldrich syndrome protein (WASP) and p21-activated kinases (PAK) (7, 8). These interactions activate the Arp2/3 complex and subsequently induce actin polymerization (9–12).

The “on” and “off” states of RhoG are constrained to two flexible loop regions: switch 1 and switch 2, which acquire conformations in the GTP-bound state that enable downstream effector proteins to recognize and interact with small Rho GTPases (13). Additionally, the intrinsic GDP/GTP switching of RhoG is slow and requires three distinct types of regulatory proteins to function, namely, guanine nucleotide exchange factors (GEFs), GTPase-activating proteins (GAPs), and guanine nucleotide dissociation inhibitors (GDIs). GEFs enhance GDP dissociation and the binding of the more abundant GTP in the cytoplasm, allowing RhoG to become active and bind to its specific effectors and hence activating signalling pathways (14). By contrast, GAPs are responsible for terminating RhoG signalling by increasing the intrinsic GTPase activity of RhoG, thereby inducing GTP to GDP hydrolysis (15). Finally, GDIs are bifunctional negative regulators required to keep RhoG GDP-bound and physically sequester it from membranes by interacting with its geranyl–geranyl group (16).

## 2 Functions of RhoG in T Cell Homeostasis

### 2.1 Role of RhoG in the Growth and Maturation of Thymocytes

The thymus is the site of T cell growth and maturation, which is critical to the sustenance of the peripheral immune system. The abnormal activities of small Rho GTPases, including RhoA (17), Rac1, Rac2 (18, 19), Cdc42 (20), and RhoH (3) are linked to thymocyte defects *in vitro* and *in vivo*. These deficiencies can be caused by defective RhoGEFs, such as Vav1, or missense mutations occurring within small Rho GTPases. For instance, loss of Vav1 in mice inhibits T cell positive and negative selection, and this process is affected by the activation status of its interacting proteins (21). Additionally, point mutations within the GEF interaction region of Rac2, such as Asp57Asn and Pro24His mutations, impair T cell development (22, 23). This finding supports the notion that proper small Rho GTPase activation is required for T cell development and maturation.

Either Rac1 or Rac2 deletion has no effect on thymocyte development, but simultaneous Rac1 and Rac2 deletions have a significant impact (18). Meanwhile, lack of RhoG has no effect on T cell formation, but it marginally increases T cell proliferation during antigen–receptor cross-linking. This finding suggests that the involvement of RhoG in T cell development is redundant compared with that of other Rac subfamily members (18), and this redundancy may be attributed to redundant signal transduction pathways (24). Both Racs and RhoG induce membrane ruffling despite their different subcellular localizations, indicating that they regulate similar signalling cascades. Nonetheless, enhanced T cell proliferation implies that RhoG has a negative impact on immune responses and its activity is crucial to the later phases of T cell activation upon antigen–receptor contact (25).

### 2.2 RhoG in Peripheral T Cell Activation

#### 2.2.1 RhoG’s Function in Proximal TCR Signalling

RhoG is involved in TCR internalization from the immunological synapse (IS) and is necessary to major histocompatibility complex (MHC) uptake in antigen-presenting cells (APCs). IS is a structured interface between a T cell and an APC, and TCR internalization at IS is required for successful T cell activation and long-term TCR engagement and signalling. However, the significance of IS in TCR activation regulation is controversial because TCR can be triggered prior to or in the absence of IS formation (26). Martínez-Martín et al. (2011) discovered that TCR endocytosis and signal extinction can occur at IS, indicating that not only IS is required to enhance TCR signalling in response to a small amount of peptide antigen-major histocompatibility complex (pMHC) ligand but also suppresses signalling by downregulating TCR in response to a high concentration of pMHC (27). The reason is that non-engaged TCRs continue to be internalized and recycled to the membrane through dynamin-dependent clathrin-mediated endocytosis (CME) in the absence of pMHC or stimulation. However, when TCRs are coupled with pMHC, their membrane expression is reduced because of enhanced TCR endocytosis, which can be regulated by CME and clathrin-independent endocytosis (28).

Martínez-Martín et al. found that RhoG enables TC21 (Rras2), a small GTPase-related to the R-RAS subfamily, to regulate TCR internalization through clathrin-independent endocytosis (26). This process may require both small G proteins to cycle between an active GTP-bound state and an inactive GDP-bound state because dominant inactive (Thr17Asn) and constitutively active (Gln61Leu) mutants cannot block TCR endocytosis. RhoG involvement in endocytosis is observed not only in T cells but also in macrophage (29) and caveolar endocytosis (30). Notably, RhoG and TC21 are associated with TCR-mediated peptide:MHC trogocytic absorption, which is needed for intercellular communication and immunological control (28). Trogocytosis is the exchange of intact membrane fragments across cells and is critical to T cell and APC activation modulation (31). Interestingly, Boccasavia et al. reported that when an antigen is introduced to naive CD4+ T cells by pMHC-II-dressed CD4+ T cells, the naive CD4+ T cells transform into pathogenic Th17 cells, and the process can be mediated by RhoG trogocytosis (32). This is because the loss of RhoG limits Th17 proinflammatory cell differentiation and promotes resistance to experimental autoimmune encephalitis development (32).

#### 2.2.2 RhoG’s Function in Distal TCR Signalling

Interestingly, immunoglobulin (Ig)G1 and IgG2b levels increase in RhoG^–^deficient mice, indicating an increase in humoral immune response to antigens (24). This finding suggests that RhoG is required for signal transduction to terminate and for the prevention of T- or B cell hyperactivation and control of autoimmunity. However, given that its subfamily member Rac2 regulates Ca^2+^ influx in response to antigen stimulation (33), RhoG may regulate Ca^2+^ influx as well, which is required for T cell-dependent immune responses and rapid cytoskeleton remodelling (34). Nonetheless, only a slight drop in Ca^2+^ influx was observed in RhoG-deficient mice upon TCR stimulation, hinting that RhoG plays a role in Ca^2+^ influx regulation (24). Notably, nuclear factor of activated T cell (NFAT) activation is dependent on Ca^2+^ mobilization, specifically through calcium–calcineurin signalling. In this signalling pathway, Ca^2+^ influx *via* Ca^2+^ release-activated Ca^2+^ (CRAC) channels is required to activate calmodulin (CaM) and the serine/threonine phosphatase calcineurin. Calcineurin then dephosphorylates serine/threonine residues in the regulatory domain of NFAT, exposing nuclear localization signals and thus promoting NFAT nuclear localization. Surprisingly, the elevation of intracellular Ca^2+^ promotes T cell anergy, a state in which a TCR becomes uncoupled from its downstream signalling pathways. This result suggests that RhoG and NFAT play critical roles in T cell tolerance induction (27).

**Figure 1:**
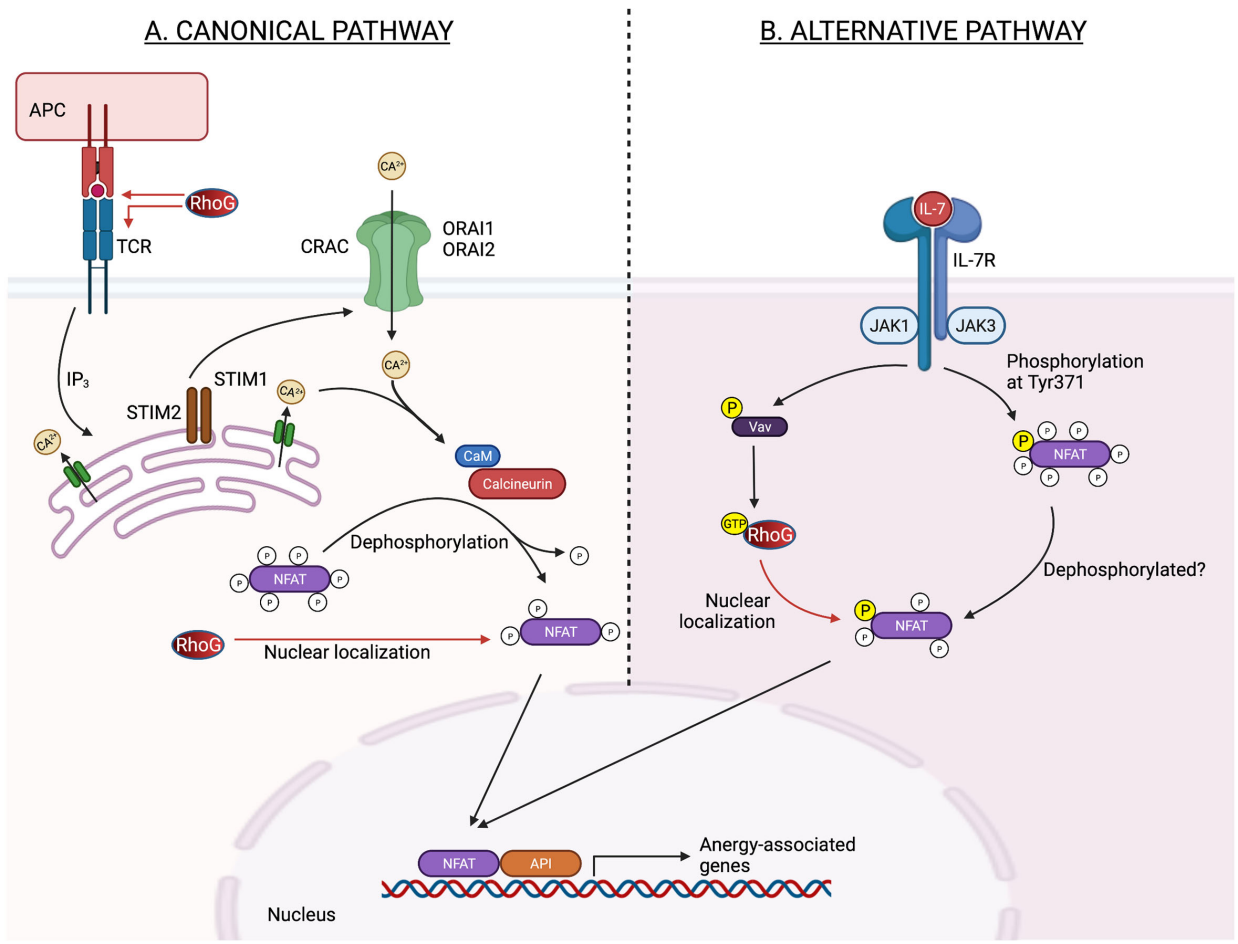
Potential role of RhoG in the regulation of NFAT transcription activity in anergic T cell in canonical and alternative pathways. **(A)** In the classical pathway, antigen receptor stimulation causes the synthesis of inositol-1,4,5-triphosphate (IP_3_), which opens IP_3_ receptor channels in the ER. The drop in ER Ca^2+^ concentration activates STIM1 and STIM2, which are needed to bind to and open CRAC channels generated by ORAI1 and ORAI2 proteins in the plasma membrane. CaM and the serine/threonine phosphatase calcineurin are then activated by Ca^2+^ inflow *via* CRAC channels. Calcineurin dephosphorylates numerous serine/threonine residues in the regulatory domain of NFAT, causing a conformational shift, nuclear localization signal exposure, and NFAT nuclear import. Increased nuclear localization of NFAT may thereby potentiate the NFAT-induced-anergy-associated gene. **(B)** Meanwhile, in an alternate pathway, Jak3 phosphorylates a single tyrosine residue within the regulatory domain of NFAT, downstream of the IL-7 receptor, causing nuclear translocation and activation of NFAT in thymocytes independent of Ca2+ signals and calcineurin. Jak3 may activate RhoG *via* Vav, causing NFAT to be localized in the nucleus. ER, endoplasmic reticulum; NFAT, nuclear factor of activated T cells; STIM, stromal interaction molecule; CRAC, Ca2+ release-activated Ca 2+; CaM, calmodulin; Jak3, Janus kinase 3; IL-7, interleukin-7. Created with BioRender.com.

Vigorito et al. discovered that RhoG can enhance NFAT/AP-1-induced interleukin (IL)-2 or interferon-gamma (IFN-γ) transcription (35), and both cytokines are related to T cell anergy (36). Numerous studies have established a link between NFAT signalling and T cell unresponsiveness or reduced responsiveness to subsequent physiological outputs, such as T cell proliferation or differentiation. This T cell unresponsiveness can be induced by inducing the transcription of anergy-associated genes, such as IL-2, IL-5, and IFN-γ or disrupting the interaction between NFAT and AP-1 (36, 37) (**Figure 1**). The latter part is predicted because RhoG contains an NLS motif at Pro179 and Ile182 residues, implying that it might regulates the activities or interactions of transcription factors (35, 38). Apart from regulating NFAT activity, RhoG may also promote T cells in a quiescent state by regulating the activities of other transcription factors, including Stat3, as RhoG promotes the transcriptional activation of Stat3 in murine fibroblasts (38). Increased Stat3 activity limits T cell proliferation by up-regulating Class-O Forkhead transcription factors (FOXO) (39). The role of RhoG in T cell anergy is supported by Martínez-Martín et al., who discovered that T cell proliferation decreases as TCR proximal signalling increases in RhoG-deficient mice (26). However, Vigorito et al. demonstrated that RhoG deficiency enhances T cell proliferation (24). Difference in T cell proliferation rate is unexpected given that TCR signalling increases. Nevertheless, these data show that RhoG is required for successful TCR signalling activation. The contradictory results observed in both studies can be explained by the fact that the doses or affinity of peptide antigens used in each research varies (40).

Additionally, RhoG may impact TCR signalling and the NFAT nuclear translocation in a calcium–calcineurin-independent manner. This process can be induced by Jak3 kinase as NFAT2 nuclear translocation is dependent on Jak3 phosphorylation upon IL-7 activation. The process then leads to the nuclear translocation and activation of NFAT2 (27). Incidentally, JAK3 is necessary for optimal Rac1 activation (41), and given that RhoG and Rac1 are 70% identical (3), Jak3 may influence RhoG activation through Vav1 (42) and affect NFAT nuclear translocation (**Figure 1**). This finding is supported by Martínez-Martín et al., who found that RhoG must be capable of cycling between active and inactive states to regulate TCR internalization and activation (26). However, Puga et al. discovered that caspase 3 cleavage inactivates Vav1 in anergic T cells (43), indicating that the RhoG activation cycle is disrupted and it may exist primarily in an inactive GDP-bound state.

### 2.3 RhoG’s Role in Controlling the Actin–Cytoskeleton and Migration of T Cells

Similar to other small Rho GTPases that are strongly associated with leukocyte transendothelial migration, RhoG is involved in the regulation of T cell migration, which requires a series of coordinated stages, complex modulation of integrin activation by chemokines, and cooperative action of adhesion molecules on endothelial cells and leukocytes (44). However, the involvement of RhoG in the control of actin–cytoskeleton complex is redundant. Nevertheless, it enhances NFAT-induced production of IFN-γ and promotes T cell recruitment to inflammatory sites (35). Additionally, the T cell production of IFN-γ is necessary for neutrophil chemotaxis to damage sites (45). Interestingly, the role of IFN-γ in the control of T cell or lymphocyte migration necessitates the modification of the expression of numerous integrins, including α4 (ITGA4), β7 (ITGβ7), and αvβ3 (35, 46, 47). For instance, upon IFN-γ stimulation, α4 and β7 expression increase, whereas αvβ3 expression decreases, and thus lymphocyte migration is promoted. These results show that RhoG has an indirect role in the control of integrin expression, as evidenced by its capacity to stimulate NFAT.

Upstream involvement of RhoG is necessary to the regulation of the activities of Cdc42 and Rac1, which are required for the production of membrane ruffles and filopodia (48). These characteristics are critical during cell migration and necessitate the participation of filamentous actin (F-actin). Reduced F-actin levels then influence the shapes of cells and the creation of force during cell migration and division. Interestingly, GTP-bound Rac1 regulates F-actin polymerization in lamellipodia (49), which may need the RhoG effector, ELMO, and the ELMO-binding protein Dock180 and Dock4, both of which are Rac1-specific GEFs (50). When RhoG is activated, the Dock-ELMO complex translocates to the plasma membrane, activating Rac1 and resulting in cell migration. This finding indicates that RhoG acts upstream to Rac1 and its activation is required for Rac1 activity, particularly cell motility. Interestingly, the absence of RhoG also inhibits RhoA activation, thereby decreasing the overall F-actin level (51). Altogether, these findings suggest the role of RhoG in T cell migration is regulating the activation of other small Rho GTPases.

## 3 RhoG Activity Is Dysregulated in T Cell-Related Disorders

### 3.1 Thymoma

According to the cBioPortal and TCGA datasets (accessed December 2021), RhoG is frequently altered by amplification, deletion, or mutation, and aberrant RhoG gene expression has been observed in various malignancies, including thymoma. Thymoma is a relatively uncommon tumour of thymic epithelial cells. Various abnormalities have been described in thymomas and affect normal T cell development by distorting tumour architecture and inhibiting MHC class II expression, autoimmune regulator gene expression, and formation of regulatory T cells (52). RhoG has been implicated in thymoma in type AB thymoma (cBioPortal) caused by a RhoG mutation at Ala151Ser.

Interestingly, RhoH Ala151Val mutation produces a loss-of-function effect, implying that a RhoG mutation at the same location may have the same effect. However, both RhoH and RhoG only share 40% sequence similarities (3), indicating that it may give a different effect. Most of the literature indicates that RhoG plays an active role in cancer progression by promoting cell migration, proliferation, and angiogenesis, and its absence is related to the reduction of cancer characteristics. Nonetheless, given the evidence of RhoG’s involvement in thymoma and lack of its function in the regulation of thymocyte development, RhoG Ala151Ser mutation may affect the activities of other small Rho GTPases, such as Rac1 and Cdc42, leading to impaired thymocytes development.

### 3.2 Hemophagocytic Lymphohistiocytosis

Hemophagocytic lymphohistiocytosis (HLH) is a potentially fatal disease characterized by a generalized inflammatory response caused by abnormal immune activation. The estimated prevalence of HLH cases in different regions worldwide varies from 1 to 10 in 1,000,000 of people. However, the reported data might have been underestimated because of scarce documentation (53–56). In general, HLH can be distinguished into primary (or familial) that is inheritable, whereas secondary HLH (predominantly endured by adult individuals) are mostly triggered by three main factors: infections, autoimmune diseases, and neoplasms (56, 57). Secondary HLH particularly induced by infection is almost similar to sepsis according to abnormal inflammatory syndrome as a consequence of infection and leads to organ dysfunction resulting from a “cytokine storm.” Given that HLH syndrome can be nearly identical to sepsis, it may unintentionally lead to the death of individuals who were misdiagnosed with sepsis (58). Mechanistically, this immunological disorder is characterized by systemic inflammation produced by the defective exocytosis of cytotoxic granules (CG), required for lymphocytes to eliminate infected or malignant cells (51).

Recently, a missense mutation, Glu171Lys in RhoG has been found to impair cytotoxic T lymphocyte (CTL) and natural killer (NK) cell exocytosis, resulting in the development of a severe HLH (51). RhoG knockout promotes deleterious effects on human NK and CD8+ T cell exocytosis as manifested by impaired cytoskeletal and cell morphology and abnormal migratory capacity (51). The data hence suggest the critical role of RhoG in CG docking to the membrane of cytotoxic lymphocytes.

## 4 Conclusions and Future Perspectives

RhoG is a critical component of T cell signalling and may be used or targeted therapeutically in cancer and immune-related diseases. However, existing understanding is insufficient and requires additional comprehensive experimental validation. Besides, inquiry into various illnesses and biological functions is also needed to enhance the knowledge of the therapeutic utility of targeting RhoG signalling axes.

The role of RhoG in thymocyte development is redundant compared with the roles of other members in the subfamily. Nonetheless, RhoG may be crucial to the control of T cell anergy through NFAT transcriptional activity or TCR endocytosis from the IS. Thus, further research into the role of RhoG in the control of T cell anergy may aid the development of therapeutic targets for the rescue of anergic T cells in human diseases, such as cancer, autoimmune disease, and viral infection. However, targeting a signalling node protein required for normal physiology is difficult, justifying the need for substantial research before identifying and designing the most effective attack points for treating RhoG-associated diseases.

## Author Contributions

All authors listed have made a substantial, direct, and intellectual contribution to the work, and approved it for publication.

## Funding

The work has been funded by the Universiti Sains Malaysia (Short-term Research Grant) No. 304/PTEKIND/6315523 granted to AMAM.

## Conflict of Interest

The authors declare that the research was conducted in the absence of any commercial or financial relationships that could be construed as a potential conflict of interest.

## Publisher’s Note

All claims expressed in this article are solely those of the authors and do not necessarily represent those of their affiliated organizations, or those of the publisher, the editors and the reviewers. Any product that may be evaluated in this article, or claim that may be made by its manufacturer, is not guaranteed or endorsed by the publisher.
